# The contribution of stigma to the transmission and treatment of tuberculosis in a hyperendemic indigenous population in Brazil

**DOI:** 10.1371/journal.pone.0243988

**Published:** 2020-12-16

**Authors:** Ida Viktoria Kolte, Lucia Pereira, Aparecida Benites, Islândia Maria Carvalho de Sousa, Paulo Cesar Basta

**Affiliations:** 1 National School of Public Health, Oswaldo Cruz Foundation, Rio de Janeiro, Brazil; 2 Departamento de Ciências Sociais, Universidade Federal de Grande Dourados, Dourados, Brazil; 3 Departamento de História, Universidade Estadual de Mato Grosso do Sul, Amambai, Brazil; 4 Centro de Pesquisas Aggeu Magalhães, Oswaldo Cruz Foundation, Recife, Brazil; Centre for Sexual Health & HIV/AIDS Research, ZIMBABWE

## Abstract

**Background:**

The Guarani-Kaiowá are Brazil's second-largest indigenous group. Average annual tuberculosis (TB) incidence rates among the Guarani-Kaiowá are nearly 400/100,000 in Mato Grosso do Sul state, ten times the national average. Although stigma is considered crucial for TB control in indigenous communities, few studies have investigated TB stigma among indigenous populations. This study sought to understand the role of TB-related stigma and perceptions of TB in maintaining hyperendemic TB transmission in the Guarani-Kaiowá communities.

**Methods:**

Various forms of stigma were explored through semi-structured interviews with 19 patients, 11 relatives, and 23 community members. Patients were identified from the registry of the healthcare service. Community members, selected by snowball sampling, were matched by gender and village of residence. Interviews were conducted in Guarani and Portuguese and later translated into English. Framework analysis was performed using NVivo.

**Results:**

Traditional beliefs of a weakening of the body allowing the disease to enter were common, but the exact mechanism of transmission was unknown. Strong community/public stigma associated TB with uncleanliness, abuse, and irresponsibility. Anticipated stigma led to significant treatment delays for fear of exclusion and losing employment. While most patients felt supported by their families, nearly all patients related experienced/enacted stigma in the community such as gossip, avoidance, and social exclusion, leading to long-lasting internalized/self-stigma. Secondary stigmatization of relatives was widespread, and blanket latent TB infection (LTBI) treatment of patients’ households was a contributing factor in treatment delay. The healthcare service unnecessarily added to stigmatization by enforcing separate utensils and sleeping arrangements for patients.

**Conclusions:**

Our findings suggest that stigma is a driver for treatment delay and continued transmission of TB in the community. The stigmatization of TB was rooted in a poor understanding of TB transmission, partly because of incorrect orientation by the healthcare service. Interventions to reduce TB-associated stigma are urgently needed.

## Introduction

Despite progress in the implementation of tuberculosis (TB) control programs, TB remains the world’s leading infectious killer [1]. Data on TB in indigenous populations is scarce but the existing evidence shows a disproportionately higher risk of TB for indigenous populations worldwide [2], even in high-income, low-risk countries [3–5]. Poor living conditions including overcrowding and insufficient ventilation, malnutrition, co-infections, and substance abuse make indigenous groups vulnerable to TB [6]. Like other vulnerable groups, indigenous populations face barriers to diagnosis and treatment including limited financial resources, living in remote areas with difficult access to healthcare, as well as discrimination and human rights abuses [6]. Although addressing stigma is considered crucial for TB control [7], little is known about the role of stigma in indigenous populations.

Brazil is a high-burden country with approximately 96,000 incident cases and 6,700 TB deaths in 2019 [1]. Brazil is home to 305 different indigenous ethnic groups, comprising around 900,000 people [8]. High TB incidence rates [9], latent TB prevalence over 40% [10], and recent and on-going transmission patterns [11] suggest that TB transmission is still hyperendemic in Brazilian indigenous territories despite free and easily accessible healthcare through the Special Indigenous Sanitary Districts (DSEI).

The Guarani-Kaiowá are Brazil's second-largest indigenous group with around 44,000 people [8]. Since the 1970’s, the Guarani-Kaiowá have re-occupied lands they traditionally inhabited, leading to violent conflict with neighboring ranchers [12]. Living on overcrowded reservations in the Brazilian state of Mato Grosso do Sul (MS), the Guarani-Kaiowá rely on farmhand contracts in an uneasy relationship with the surrounding plantations [13].

The disadvantaged living conditions of the Guarani-Kaiowá, characterized by extreme poverty [14], food insecurity [15], and high levels of violence [16], favor transmission of TB. A 2013 study showed that the indigenous population of Mato Grosso do Sul had a relative risk of TB 7.3 times higher than the state average. The Guarani-Kaiowá population in the district of Amambai showed markedly higher incidence rates than the rest of the state, with rates of diagnosed active TB up to 400/100,000, ten times the national average [17].

Taking these points into account, the aim of this study was to understand the role of TB-related stigma and perceptions of TB in maintaining hyperendemic TB transmission in the Guarani-Kaiowá communities to inform design of interventions.

## Materials and methods

### Study setting

The district of Amambai, MS, on the border with Paraguay, manages several smaller healthcare stations located in the local indigenous territories as first points of care. The population of these territories is almost exclusively of Guarani-Kaiowá ethnicity. The study took place in four indigenous territories in the catchment area of Amambai; *Amambai* (population: 8610), *Limão Verde* (population: 1810), *Taquapery* (population: 3466), and *Guassuty* (population: 669).

### Study tools development

Based on previous fieldwork observations, we developed two interview guides for assessing perceptions of TB transmission and understanding stigma from different levels and from the viewpoints of patients, relatives, and community members. One interview guide was developed for TB patients (TB Cases, TBCs), (S1 Appendix), and another for Community Members (CMs) who had not had TB, (S2 Appendix). In collaboration with the organization Young Indigenous in Action (Portuguese acronym JIGA) in Amambai, the interview guides were tested and revised to ensure that the questions were appropriate, culturally sensitive and formulated using a Guarani-Kaiowá way of expression to avoid that the essence of the questions would be lost in translation from Portuguese to Guarani.

### Data collection

Six (n = 3 male, n = 3 female) Guarani-Kaiowá research assistants from the JIGA group received training during the week prior to data collection in the form of a series of work group sessions, led by IVK, LP, and AB, where the best translations of the questions, methods of approaching interview participants, interview techniques, the concept of informed consent, and transcription and translation were discussed and agreed upon. All six research assistants were university graduate students aged 19–23 years, and were familiar with the overall research project *“Social Inequalities and TB*: *Transmission Dynamics*, *Living Conditions and Interfaces between Biomedicine and Traditional Indigenous Medicine”* [14] from participation in previous fieldwork as interpreters and focus group moderators. The research assistants played a key role as ‘‘ambassadors” in the community, making introductions and interpreting when interview participants preferred speaking in Guarani.

TB patients (TBCs) who had been treated for TB during the period January 2014-May 2016 were identified from the Amambai district registry, working backwards from the most recent cases. Mindful of close Guarani-Kaiowá family structures, we did not insist on strictly individual interviews if the participant wanted to be interviewed with family members present. Adult members of TB patients’ households present at the interview were invited to participate (TB Patient Relatives, TBRs), and were interviewed using the same interview guide as the CMs. CMs were selected by snowball sampling and matched by gender and indigenous territory of residence. The semi-structured interviews took place in the home of the interview participant and were conducted in Guarani or Portuguese depending on the interviewee’s preference. For interviews conducted in Guarani, LP, AB, or a research assistant would ask the questions and act as simultaneous interpreter to IVK. Respecting traditional gender divisions, the researchers IVK, LP, and AB were accompanied by gender-specific research assistants. All interviews were audio-recorded. Data saturation was reached when no new comments were appearing in the interviews, as described by Fusch & Ness [18].

### Data analysis

Audio-recorded data, transcribed and translated from Guarani to Portuguese (where necessary) by the research assistants under the supervision of LP and AB, were analyzed using framework analysis. After an initial review of the interviews and field notes by all authors, IVK, IMC, and PCB separately coded passages of interest in the interviews. IVK drafted the first thematic framework which was compared with the coding of IMC and PCB. We generally highlighted the same passages but discussed the coding labels relating to the sources/actors in anticipated stigma until consensus was reached. The draft framework was then discussed with the JIGA group to minimize bias by balancing views from inside and outside the community. Using the finalized thematic framework, IVK undertook the coding using NVivo (QSR International, Melbourne, Australia) software and drafted the first analysis of the passages for each theme for revision by the other authors (S3 Appendix). Citations from the interviews in this text were translated from Portuguese to English by IVK and revised by the other authors.

Throughout the text findings are presented according to the following scale of representation of interviewees: ***few***, <20%; ***some***, 20–40%; ***many***, 40–60%; ***most***,60–80%; ***nearly all***, 80–100%.

### Ethical considerations

This study is part of the project *“Social Inequalities and Tuberculosis*: *Transmission Dynamics*, *Living Conditions and Interfaces between Biomedicine and Traditional Indigenous Medicine”*, approved by the Research Ethics Committees at the National School of Public Health at the Oswaldo Cruz Foundation (FIOCRUZ) (#15988913.9.0000.5240/354.060), and the Ministry of Health (#15988913.9.0000.5240/650.820). Written informed consent was obtained from all participants. Participants who did not speak Portuguese would have the consent form orally translated and explained in Guarani. Participants unable to read and write would have the form read to them by a relative or one of the Guarani-Kaiowá research assistants and sign with a fingerprint. No honorarium was offered for the interview.

## Results

The interviews were carried out in June 2016. Data were saturated with 53 interviews, with a mean length of 54 minutes. Four male CMs from the Guassuty indigenous territory declined to interview as they were on their way to another location. In five households of TB patients, other family members were at home but did not come out to meet us. We interviewed 19 TBCs, 11 TBRs (from 11 different households), and 23 CMs, Table 1. It was our experience that the presence of family members made for richer interviews as participants felt more at ease talking to us. No stigmatization within the household was reported in households where both a TBC and a TBR were interviewed.

**Table 1:** Participants by category, gender, and village of residence.

	Men	Women	Total
**Total interviews**	24	29	53
**Cases (TBCs)**	7	12	19
**Relatives (TBRs)**	6	5	11
**Community members (CMs)**	11	12	23

We present qualitative findings according to the standardized TB stigma categories developed by the Challenge TB Stigma Measurement Guidance [19]: a) *community/public stigma*; negative attitudes to TB in the community, b) *anticipated stigma*; the expectation and/or fear of being stigmatized, adopting behavioral changes, c) *experienced/enacted stigma*; concrete experiences of exclusion/discrimination, seen either from the viewpoint of the stigmatized (experienced stigma) or the stigmatizer (enacted stigma), d) *internalized or self-stigma*; changes in self-perception, loss of self-esteem and/or feelings of guilt or shame as the individual has come to adopt negative stereotypes, and e) *secondary stigma*; stigmatization by association with a TB patient.

### Perceptions of TB transmission

All participants correctly described TB symptoms including coughing, chest pain, and weight loss, and had a clear concept of TB as a transmissible disease. Participants reported a range of TB transmission beliefs, but no participant had a biomedical understanding of TB as an airborne respiratory bacterial infection. Central to the understanding of TB transmission was the notion that the disease can take hold if the body is weakened, although the exact mechanism was not clear. Nearly all participants identified sudden weight loss with active TB. Being fat was associated with wealth and/or health: *‘‘Back then*, *there were the sugarcane factories*. *Back then there was money*. *Everybody was fat*!*” (CM6)*. Many participants described the weakening of the body as a result of malnutrition: *‘‘Children get TB because they do not get anything to eat (*..*) Even we adults sometimes go hungry and then we get it [TB] too” (CM5)*. Alcohol/drug abuse and smoking featured in many interviews as the cause of TB as users would not feel hunger and/or not have the money to buy food, thus exposing the body to TB infection: *‘‘You get TB because of hunger*. *If somebody is drinking*, *they drink all day long and stay hungry all day*, *the children go hungry because there is no food in the house” (CM13)*. Resonating the theme of food scarcity, a few participants reported eating decomposing/unclean food as a vehicle for acquired TB: *‘‘Many people go into town with their children and go through the trash to find something to eat” (CM5*).

Further, many participants reported that living in dirty, dusty, and/or bug-infested environments increased the risk of TB; *‘‘Many of our women do not take care of their houses*. *They leave everything dirty—they do not clean around the house*. *TB comes more quickly when things are dirty” (CM8)*. Contact with dirty hands or with contaminated utensils such as spoons, plates, and cups was believed by many CMs to cause TB: *‘‘This disease is really infectious*. *It spreads via spoons” (CM9)*. Few TBCs and TBRs expressed this view. Some participants referred to the working conditions on the sugarcane plantations which involve exposure to dust and ashes: ‘‘*I worked on the sugarcane plantation*, *burning the cane [to facilitate the harvest]*. *There were a lot of ashes and that stuck in my lungs” (TBC10)*.

Many participants expressed beliefs of TB transmission rooted in traditional Guarani-Kaiowá concepts of disease as an imbalance between body and soul, leaving an opening for evil spirits to bring in disease. Being afraid of the disease, or of contact with a TB patient, can be a weak spot through which the spirit of the disease can enter: *‘‘If you look at the body of a person who died and had TB*, *and you are afraid*, *then the disease will come to you” (CM4)*. Some participants reported that diseases or witchcraft may be in the air at certain times, during which it is recommended to stay indoors to avoid TB: ‘*My daughter told me ‘‘be careful when you go outside because the diseases are around right now”“(TBC2)*.

A few participants saw TB as a result of accidents or violence where the disease enters the body through injuries: ‘*‘Her ex-husband gave it [TB] to her*. *He raped her and put a bottle up her vagina and left her for dead*. *He put the disease inside her”* (TBR2).

### Community/Public stigma

Most CMs expressed views that a TB patient must be socially isolated while on treatment, with many believing that this isolation must include the entire household of the TB patient. *‘‘When one has TB*, *obviously everybody in that house should be treated and keep a distance so they do not spread it to others”(CM9)*. Most CMs stressed that a person with TB contaminates cutlery and plates; *‘‘If I have TB and I eat*, *and somebody else eats from that plate*, *then he already gets it (CM16)*. Many CMs thought that the isolation could be lifted when the infected person had completed treatment.

Describing how people with TB symptoms would delay seeking treatment, many CMs condemned them for putting others at risk: *‘‘They go to the doctor only after somebody looks at that person and sees that he or she is very thin and asks if they feel any pain*, *and even then they will say no*. *I think it is lack of respect*, *because that person must know that the disease is attacking him or her” (CM12)*. Many CMs also perceived people with tuberculosis as unable or unwilling to take care of themselves, thus letting the disease progress and subsequently transmitting it to others: *‘‘It is not like I want to abandon my friend if he has TB*, *but there is also a part where you need to make an effort and go to the doctor and take care of yourself*, *because that disease is passed on from one to another” (CM20)*. This perceived lack of willingness to take control of the disease was stated as the reason for isolating oneself from people with TB, thus holding the patient accountable for infecting others; *‘‘In the sugarcane plantations there is already a lot [of TB]*. *But it is here in the village that you get it because these people do not take care of themselves” (CM9)*.

### Anticipated stigma

Anticipating rejection, most TBCs related that they only told the nearest family and the community healthcare agent about their TB diagnosis; *“After I got TB*, *I did not leave the house anymore*. *That way no one would find out*. *Only the nurses knew about it” (TBC18)*. Anticipating ostracization from the community, some TBCs related not going to the healthcare station for a consultation as they did not want to be seen receiving the directly observed treatment in front of their house every day.

Most male TBCs, employed by the sugarcane plantations in the regions on fixed-term contracts when they felt the first symptoms, would try to hide their illness until the end of the contract, sometimes for several months, as they did not want to be sent home and lose the work: *‘‘I was on the sugarcane plantation and I was feeling ill for a long time*. *I could work only a little before I could no longer breathe*. *For 6 months I worked like that*. *One day I had to sit down*, *and the foreman saw me*. *He took me to the hospital*, *and they told my wife that I had died” (TBC18)*.

### Experienced/Enacted stigma

Most TBCs related one or more concrete experiences of stigmatization. Where TB was discovered while the TBC was working on a sugarcane plantation, the response varied considerably by plantation. Some TBCs related being sent back to work after a superficial examination at the workplace, some were sent home, a few were taken to the healthcare service, and a yet another few reported receiving an examination by an external healthcare provider.

While a few TBCs said that a worker could come back to work after having TB, some TBCs related personal difficulties with finding employment after having completed TB treatment as employers were uninterested in workers with a history of TB and could afford to turn them down as the supply of workers was much greater than the demand. *‘‘After I was cured*, *I lived like this*. *The company did not want to hire me*. *Because I was very thin*, *they did not trust me*. *‘‘You will not be able to stand it”*, *they said” (TBC11)*. Some participants reported that they were no longer able to perform the physically demanding manual labor after having had TB and had not returned to work.

Being abandoned by the family was reported by a few TBCs: *“After I got this TB*, *they [ex-wife and children] no longer wanted to stay with me” (TBC14)*. However, most TBCs experienced practical and emotional support from their immediate family members during treatment. This support was commonly seen as vital for the patient’s recovery but also as carrying great personal risk of infection for the family members. Support was extended only to close family members and/or within the household: ‘‘*Only my daughter would come*, *and she would eat and drink tererê* [iced yerba-mate drunk from a shared gourd] *with me*. *She would also let the grandchildren drink tererê with me” (TBC13)*. Some interviews with CMs corroborated that point: *‘‘If a relative of mine has TB I will avoid them*. *If we do not live together*, *I do not know if he or she is doing the treatment” (CM18)*. Experiences of avoidance and exclusion by family living in other households and by the community were not receiving visits, friends and family not allowing their children to visit, being the victim of gossip, and not being invited to share a *tererê*. One man described an episode of feeling rejected by his family living in another village: ‘*‘I went to my mother’s house (…) even she did not want to drink tererê with me because she was afraid she would pass on the disease to my brothers” (TBC16*).

Separating plates, mugs, and cutlery for TB patients is not part of the Brazilian tuberculosis control guidelines [20], but many TBCs described how staff from the healthcare service had handed out special utensils and recommended that the patient sleep and eat alone, thus unnecessarily contributing to the stigmatization of tuberculosis: *‘‘they prohibited me from drinking tererê with friends who are in good health*. *I ate lunch alone*. *I had to have a separate plate*, *separate spoon*, *and my cup had to be separate”(TBC2)*; *‘‘They told me I had to wear a mask*. *I wore the mask at home*. *I did not want to leave the house wearing that”(TBC12)*. Some CMs related how they had employed this infection control measure with relatives with TB; *‘‘Yesterday we told her that we would not eat together with them (*..*) Just by using the same spoon the disease spreads*, *and if you or your spouse has it*, *it will pass on to others who do not have it” (CM17)*. We described under perceptions of TB transmission that many CMs, but only few TBCs and TBRs, believed that TB is transmitted through contaminated/unclean cutlery and plates.

Religious communities (traditional Guarani-Kaiowá prayer houses or evangelical churches) play a central role in the social life in the territories. For some TBCs, they offered a place of acceptance and support, based on the belief that faith would help cure the patient and prevent transmission to the congregation:”*They prayed for me*. *They were not afraid*. *It [TB] will not even be passed on*, *they said”(TBC3)*. However, some TBCs experienced exclusion from their religious community: *‘‘After she got TB*, *she stopped singing in the house of prayers*. *They were afraid it would spread to the others”(CM1)*.

As in the CM interviews, many TBCs reported that the stigmatization commonly lasted only until the completion of treatment after which social relations resumed. However, a few TBCs related that TB-associated stigma persisted even after the completion of treatment: *“Some people are afraid of me and when I arrive*, *they will not drink tererê with me*. *I know they know I had TB and they are afraid of getting it*. *It is still like this [a year after]”(TBC11)*.

### Internalized/Self-stigma

Many TBCs described how the negative feelings from experienced and anticipated stigmatization in the form of rejection from family members and isolation and estrangement from the community were internalized, affecting their self-worth: ‘*‘When I went to work in the sugarcane plantation*, *I was not thin*, *I worked hard*. *When I see my brothers strong like that*, *and I am not*, *I feel strange*. *I sit here*, *I do not work*, *and I feel wrong*. *All my brothers go to work and I want to go*. *I cannot work anymore; I am no longer able to [get] work after I had TB”* (TBC11). For some TBCs, these negative emotions of rejection and isolation led to suicidal thoughts: ‘*‘My whole family was afraid of me and it made me feel rejected*. *I am afraid of visiting people*. *When I arrive*, *sometimes I feel that they will talk about me; ‘‘Look who came*, *she has TB*, *we do not want to drink tererê with her”*. *It is so difficult to see even your mother be afraid of you*. *It makes you think it would be better if you died”*(TBC12).

### Secondary stigma

Stigmatization of TB also included members of the patient’s household. Some TBRs described how they would not talk to others about the diagnosis as they anticipated rejection. These participants also related experienced stigma, where nobody wanted to touch them or be near them, and where they would be looked at from a distance: *‘‘After he got TB*, *I also stopped going to church*. *People did not like that I was there”(TBR11)*. Receiving prophylactic latent TB treatment also carried stigmatization for TBRs; *‘‘They think I have the disease already*, *and they started to distance themselves from me and talk about me that I have TB even if I do not have it”* (TBR7).

Summing up the points made in the interviews, this statement from a CM acknowledges that community/public stigmatization is ‘damaging’ to the TB patient and his/her family. Anticipating stigmatization, and knowing that his/her family will be the victims of secondary stigma, TB patients delay seeking treatment: *‘‘It is only when the disease is very advanced that they will say something*. *Because when a person gets treatment*, *their whole house needs to get treatment*. *When you say that you feel pain*, *you are damaging your entire family”* (CM17).

## Discussion

We conducted a study to explore TB-associated stigma and its impact on patients and their relatives in a Brazilian indigenous population with hyperendemic transmission of TB. We found that stigma impacts the lives of Guarani-Kaiowá TB patients and their relatives to a considerable degree, and that stigmatization represents an important barrier to treatment in this population. Patients, relatives, and community members alike held beliefs that disease is caused by a weakening of the body and spirit. The poor living conditions of the Guarani-Kaiowá were reflected in the numerous mentions of malnutrition as a risk factor.

Significant community/public stigma surrounds TB in this population, associating TB with negative behavior such as being unclean/contaminated, abusing alcohol and drugs, and/or unable to provide for oneself, leaving TB patients and their families as undesirables in the community. With an unclear understanding of the mechanism of TB transmission, some community members held TB patients personally responsible for spreading the disease in the community. Remarkably similar descriptions of anticipated and community/public stigma show that TB-associated stigma is well known in the community. The question of blame illustrates the relevance of anticipated stigma as a separate category in unpacking TB stigmatization.

Misguided recommendations from the local healthcare service to separate plates and utensils for TB patients contributed to the community/public and experienced/enacted stigmatization of TB. That a TB patient is no longer contagious after a few weeks of treatment was not discussed in any of the interviews, perhaps pointing to another gap in knowledge.

There is limited evidence on the effect of interventions reducing TB stigma [21]. While awareness campaigns are necessary, some studies note that they may contribute to the stigmatization of TB and are not sufficient in themselves to combat TB stigma [22, 23]. Language used in awareness campaigns for airborne infectious diseases tends to stigmatize patients as a risk to the community [24, 25]. Our findings point to a need for raising awareness of TB transmission dynamics in the community and among healthcare service staff as a first step towards reducing TB stigma. As most interview participants in this population did not use a biomedical model to explain disease, an awareness campaign must be sensitive to traditional views of disease transmission. Using an approach that integrates biomedical principles of TB infection and transmission with traditional concepts of disease and health has been successful in other settings [26–28].

The sugarcane plantations employing Guarani-Kaiowá men have been identified as likely TB transmission hotspots [13]. Anticipated stigma led to significant delays in seeking treatment for Guarani-Kaiowá plantation workers as they feared losing their jobs. Delaying treatment for several months increases the likelihood of the worker infecting others, and suffering permanent lung damage, affecting future employment. Interventions are urgently needed to facilitate the return to employment for workers after TB treatment. Examples could be adult learning programs, skill upgrading, government involvement in regulations and enforcement of working conditions. Inequalities including limited access to education, land, and subsequently employment opportunities, are the origin of TB transmission in these communities and are part of the discrimination and violation of the human rights of the Guarani-Kaiowá [29].

Our findings suggest that the Directly Observed Treatment, Short-course (DOTS) scheme may inadvertently cause anticipated, experienced/enacted, and secondary stigma for patients and their relatives as the healthcare worker visits to the patients reveal their TB/LTBI status to the community. Research from Amambai and its neighbor district, Dourados, has shown that the number of contacts needed to treat to prevent one additional case was 10 (CI: 6–19) for contacts with a tuberculin skin test (TST) reaction ≥10mm (38% of contacts) [30] significantly lower than a recent average global estimate of 100 [31]. With irregular TST supplies, the healthcare service in the study area gives all household contacts LTBI treatment without prior TST testing. In Amambai only around 60% of contacts are evaluated because of the high mobility of this population, particularly men employed in plantations away from home [32]. Recent genotyping analysis of TB isolates from Dourados showed that transmission occurred outside the household [33], pointing to a need for better contact tracing. Based on our findings, contact tracing strategies need to consider not only the transient household structure but also the influence of secondary TB stigmatization in this population.

While most patients experienced support from their household, patients, relatives, and community members gave examples of experienced/enacted stigma including avoidance, gossiping, exclusion from religious communities, and sharing meals and joining in for a *tererê*. Although most participants related that this social exclusion would last until the completion of TB treatment, the internalized/self-stigma of lowered self-esteem persisted. Interventions to build empowerment of patients and their relatives have shown promising results elsewhere [23, 24]. So-called stigma clubs that provide a forum for patients and their families could be a suitable intervention in this setting [34].

The conceptualization of stigma is increasingly recognized as similar to discrimination, both rooted in broader political, historical, and cultural power structures, inter- and intrapersonal attitudes to a given population group, and perceptions of health and disease [35–37]. TB patients belonging to marginalized groups often suffer social exclusion and discrimination, potentially compounding the effect of TB stigma and raising the barrier to care [19]. The International Labour Organization has reported intersectional stigma associated with being indigenous and living with HIV and/or TB in Europe, Africa, Australia, North and South America [38]. In a study of high-risk populations in Canada, Gibson et al (2005) found that TB stigmatization was more common in indigenous communities than in immigrant groups (14% versus 3%). For indigenous participants, stigmatization of TB was related to current and past experiences of discrimination of indigenous people, with past forced removals to TB sanatoria still affecting the perception of TB treatment [39]. Our results show that TB-associated stigma has an important impact on TB control in this indigenous community, and that interventions aimed at eliminating the systemic discrimination suffered by this population are urgently needed to reduce TB transmission. Isolation of indigenous TB patients in sanatoria in MS was replaced by decentralized treatment only in recent decades. Our findings suggest that more work needs to be done towards more culturally sensitive healthcare, and that more research is needed on the stigma and power structures in the relationship between the healthcare service and indigenous populations in Brazil.

To our knowledge, our study is the first detailed study of TB-associated stigma in an indigenous population in a high-burden country, and the first public health study to employ a community-based participatory research (CBPR) model in an indigenous population in Brazil. CBPR collaborations raise questions on power dynamics between external researchers, community researchers and interview participants [40, 41]. Developing the interview instruments in collaboration between non-indigenous and indigenous researchers and using Guarani as the interview language acted as a leveler of power. The young age of LP, AB, and the JIGA members also contributed to putting interview participants at ease as they were considered equals by the younger participants and non-superior by the older participants. IVK may have enjoyed a neutral position being a foreigner without ties to the Brazilian social and health services. As a woman, and also speaking Portuguese as a second language, may have contributed to the acceptance of IVK into the homes of the interview participants as she was perceived as a less dominant representative of non-indigenous society. We found that the CBPR model shows promise for working towards filling the data gap on TB and a better understanding of the intersectional stigma in indigenous populations.

Our study only included patients who had received biomedical TB treatment from the healthcare service. We do not know if, and possibly how many, TB patients choose to get treatment exclusively from traditional healers. A study of healthcare performance in a neighboring Guarani-Kaiowá territory described how the concept of a Likert-scale questionnaire was poorly understood [42]. Formally validating a quantitative research instrument was further hindered by the scattering of the Guarani-Kaiowá in small and distant territories. During previous fieldwork, we found that the organization of Guarani-Kaiowá society in closed clan entities makes focus groups unsuitable for stigma studies in this setting. We therefore chose semi-structured interviews for data collection. Triangulation of the data was made possible by interviewing patients and their relatives as well as community members who had not been personally affected by TB.

The interviews were translated from Guarani to Portuguese, and into English for the excerpts in the published text. Although great care was applied to ensure as accurate a translation as possible, minor discrepancies may have occurred where differing syntax obstructs literal translation. However, as authors LP, AB, and IVK discussed each interview in detail immediately after its conclusion and during the translation process to clarify the exact meaning of what was said, we believe to have reduced the impact of translation on our interpretation of the data as much as possible.

## Conclusions

TB-associated stigma is an important barrier to seeking treatment and a factor in the continued transmission of the disease in the Guarani-Kaiowá communities. We identified a need for awareness campaigns about TB transmission in the community and among the healthcare service staff. Interventions such as stigma clubs to empower patients and their families are urgently needed to reduce stigmatization. These culturally adapted interventions must be developed in collaboration with community members and consider the role of traditional medicine in the community. Anticipating stigma and with limited opportunities for employment, Guarani-Kaiowá workers would delay treatment. Elimination of TB in this hyperendemic population is unlikely to be achieved without addressing the violations of the human rights of the Guarani-Kaiowá. The epidemiological and economic impact of the COVID-19 pandemic on indigenous communities in Brazil is severe [43], and presents an additional challenge to TB control in the Guarani-Kaiowá communities both in terms of potential further stigmatization of airborne diseases as well as the transfer of funds from TB control to the combat of COVID-19 [44].

## Supporting information

S1 Appendix Interview guide for TBCs. Portuguese and English version.
(DOCX)

S2 Appendix Interview guide for TBRs and CMs. Portuguese and English version.
(DOCX)

S3 Appendix Thematic framework for analysis. (DOCX)
