# Supplementary material for: The contribution of stigma to the transmission and treatment of tuberculosis in a hyperendemic indigenous population in Brazil
Source: PLoS One. 2020 Dec 16;15(12):e0243988. doi: 10.1371/journal.pone.0243988 (PMC7743939; doi:10.1371/journal.pone.0243988)
Supplement: S1 Appendix — Portuguese and English version. (DOCX) [file pone.0243988.s001.docx]

**S1 Appendix. Interview guide for TBCs. Portuguese and English version.**

**Roteiro de entrevistas – estigma**

**Pacientes**

***Público-alvo: Entrevistas individuais com homens e mulheres das aldeias Amambai, Limão Verde, Taquapery e Guassuty, que foram diagnosticados com TB nos últimos 12-24 meses (por favor, refere à lista dos pacientes e começa com o paciente diagnosticado mais recentemente)***

1. ***Entendimento da doença:***

Você sabe de onde vem a TB? Se sim, você pode nos falar um pouco de onde vem a tuberculose?

Como uma pessoa adoece com tuberculose?

Porque você acha que pegou (ficou doente de) tuberculose?

Quais foram os principais sintomas que você apresentou?

O que você pensou quando teve os primeiros sintomas?

Qual foi a sua primeira reação aos sintomas?

1. ***Serviço de saúde:***

O que você fez quando começou a sentir os sintomas de tuberculose?

Você procurou ajuda de alguém?

Pensou em procurar rezador? Porque sim? Porque não? Como foi sua experiência com o rezador*? *(*Só se não mencionar isso)*

O que levou você a procurar o serviço do posto? *(Lembrete: todos eles foram atendidos pelo serviço biomédico, é assim que identificamos eles como casos)*

Como você foi recebido ou tratado pelos profissionais de saúde no posto? Quem foi que o atendou? Médico? Enfermeiro? Técnico de Enfermagem? AIS?

Como foi o relacionamento com a equipe do posto durante seu tratamento?

Você pode nos contar um pouco sobre sua experiência com seu atendimento no posto, durante o tratamento para TB?

1. ***Stigma***

Como você se sentiu ao saber que estava com TB?

Alguma coisa mudou em casa depois que você descobriu que tinha TB?

Como se sentiu quando falou para sua família que estava com TB?

Como as pessoas da sua família reagiram? Você teve algum problema com sua esposa(o) ou filhos? Você pode nos contar que tipo de problema você enfrentou?

Você teve que deixar o trabalho depois que ficou doente com TB? Se sim, porque? Se não, como você conseguiu trabalhar estando doente? Como foi no seu trabalho depois que foi diagnosticado com TB?

Como seus amigos e parentes reagiram quando falou para eles que estava com TB? Eles te apoiaram ou se afastaram de você? Você pode nos contar como foi essa experiência?

Você frequenta alguma igreja/casa de reza? Como foi sua participação na igreja/casa de reza depois que você descobriu que estava como TB?

Sua vida mudou em alguma maneira depois que você teve TB? Se sim, você pode nos contar como foi?

Você mudou algumas atitudes ou hábitos depois que descobriu que teve TB? Pode nos contar sobre isso?

Sua percepção sobre você mesmo mudou de alguma maneira depois que teve TB? Você pode nos falar mais sobre isso?

Vamos terminando por aqui, mas antes gostaria de saber se você deseja falar mais alguma coisa sobre TB que não discutimos?

**Interview guide – stigma**

**Patients**

***Target audience: Individual interviews with men and women from the indigenous territories of Amambai, Limão Verde, Taquapery, and Guassuty, who have been diagnosed with tuberculosis in the last 12-24 months (please refer to list of patients and begin with the most recently diagnosed).***

1. ***Understanding of the disease:***

Do you know where tuberculosis comes from? Can you tell us a little about where tuberculosis comes from?

How does a person become ill with tuberculosis?

Why do you think you got tuberculosis?

What were the main symptoms that you felt?

What did you think when you felt the first symptoms?

What was your first reaction to the symptoms?

1. ***Healthcare service:***

What did you do when you started to feel the symptoms of tuberculosis?

Did you ask somebody for help?

Did you think about seeking help from a traditional healer? If yes, why? If no, why not? How was your experience with the traditional healer*? *(*if the person does not mention this himself/herself)*

What made you seek help from the healthcare station? *(NB: All the cases on list were treated by the healthcare station, that is how we identified them as cases)*

How were you received by the healthcare professionals at the healthcare station? Who looked after you? Was it medical doctor? Nurse? Nursing assistant? Indigenous Healthcare Assistant?

How was your relationship with the healthcare team at the station during your treatment for tuberculosis?

Can you tell us a little about your experience with the healthcare you received at the healthcare station during your treatment for tuberculosis?

1. ***Stigma***

How did you feel when you discovered you had tuberculosis?

Did anything change at home after you discovered you had tuberculosis?

How did you feel when you told your family that you had tuberculosis?

How did your family members react? Did you have any problems with your spouse or other family members? Can you tell us a little about the problems you had to face?

Did you have to stop working after you got tuberculosis? If yes, why? If no, how did you manage to work while you were ill? How was your experience at work after you were diagnosed with tuberculosis?

How did your friends and relatives react when you told them that you had tuberculosis? Did they support you or did they distance themselves from you? Can you tell us a little about your experiences and how this made you feel?

Do you go to church or the prayer house? How was your experience in the church/prayer house after you discovered you had tuberculosis?

Did your life change in any way after you discovered you had tuberculosis? If yes, can you tell about that?

Did you change any attitudes or habits after you discovered you had tuberculosis? Can you tell us more about that?

Did you see yourself in a different way after you discovered you had tuberculosis? Can you tell us more about this?

These were our questions about tuberculosis. Is there anything else that you would like to say about tuberculosis?
