# Supplementary material for: The contribution of stigma to the transmission and treatment of tuberculosis in a hyperendemic indigenous population in Brazil
Source: PLoS One. 2020 Dec 16;15(12):e0243988. doi: 10.1371/journal.pone.0243988 (PMC7743939; doi:10.1371/journal.pone.0243988)
Supplement: S2 Appendix — Portuguese and English version. (DOCX) [file pone.0243988.s002.docx]

**S2 Appendix. Interview guide for TBRs and CMs. Portuguese and English version.**

**Roteiro de entrevistas – estigma**

**Não-pacientes**

**Público-alvo: Entrevistas individuais com homens e mulheres das aldeias Amambai, Limão Verde, Taquapery e Guassuty, que não foram diagnosticados com tuberculose.**

1. ***Entendimento da doença:***

Você sabe de onde vem a tuberculose? Você pode nos falar um pouco de onde vem a tuberculose?

Como uma pessoa fica doente com tuberculose?

Como se reconhece/identifica/sabe que uma pessoa está com tuberculose?

Você conhece alguém que ficou doente com tuberculose?

1. ***Serviço de saúde/tratamento:***

Você pode nos falar como deve ser tratada a tuberculose?

Qual é a melhor maneira de tratar TB? Quem sabe tratar melhor a tuberculose? Por quê?

Quais são as diferenças entre o tratamento do rezador/rezadora e o tratamento do posto de saúde?

O que você faria se tivesse tuberculose? Por quê você faria essas escolhas e não outras?

1. ***Estigma/preconceito na comunidade:***

Se uma pessoa estiver com tuberculose, como ela vai ser tratada da comunidade? Você pode nos explicar melhor e dar alguns exemplos? Por quê isso acontece?

O que acontece na família se alguém está com tuberculose?

O que acontece no trabalho se alguém está com tuberculose?

Você frequenta alguma igreja/casa de reza? O que acontece na igreja/casa de reza se alguém está com tuberculose?

Como você deve se comportar com uma pessoa que está com tuberculose?

Como uma pessoa que está com tuberculose deve se comportar? O que a pessoa com tuberculose tem que observar? Ela ou ele precisa fazer alguma coisa diferente do restante da comunidade? Por quê? Quais cuidados ela ou ele deve tomar?

1. ***Experiência pessoal:***

Você conhece alguém que teve tuberculose? Você pode nos contar um pouco como foi o convívio com essa pessoa?

Como você reagiu quando descobriu que ele ou ela teve tuberculose?

Sua vida mudou porque alguém que conhece teve tuberculose? Se sim, pode nos contar como foi?

Como você se sentiu em relação à essa pessoa? Como se sente agora?

Você mudou alguns pensamentos ou comportamentos desde que descobriu que essa pessoa estava com tuberculose?

Vamos terminando por aqui, mas antes gostaria de saber se você deseja falar mais alguma coisa sobre tuberculose que ainda não discutimos?

**Interview guide – stigma**

**Non-patients**

***Target audience: Individual interviews with men and women from the indigenous territories of Amambai, Limão Verde, Taquapery, and Guassuty, who have never been diagnosed with tuberculosis.***

1. ***Understanding of the disease:***

Do you know where tuberculosis comes from? Can you tell us a little about where tuberculosis comes from?

How does a person become ill with tuberculosis?

How can you tell that a person has tuberculosis?

Do you know anybody who had tuberculosis?

1. ***Healthcare service and treatment***

Can you tell about how tuberculosis should be treated?

What is the best way to treat tuberculosis?

Who knows best how to treat tuberculosis? Why?

What are the differences between the treatment offered by the traditional healer and the treatment at the healthcare station?

What would you do if you had tuberculosis? Why would you make these choices and not others?

1. ***Stigmatization:***

If somebody has tuberculosis, how will he or she be treated by the community? Can you tell us a little more about this and give some examples? Why do these things happen?

What happens in the family when somebody has tuberculosis?

What happens at work when somebody has tuberculosis?

Do you go to church or the prayer house? What happens in the church/prayer house if somebody has tuberculosis?

How should you act around somebody who has tuberculosis?

How should a person with tuberculosis act? What should a person with tuberculosis do? Does he or she need to do something different than the rest of the community? Why? What are the measure that he or she must take?

1. ***Personal experience***

Do you know anybody who had tuberculosis? How was it being around that person?

How did you react when you discovered that he or she had tuberculosis?

Did your life change in any way because somebody you know had tuberculosis? If yes, can you tell us more about that?

How did you feel around that person? How do you feel now?

Did you change your mind or your behaviour after you discovered that he or she had tuberculosis? If yes, can you tell us more about that?

These were our questions about tuberculosis. Is there anything else that you would like to say about tuberculosis?
