# Supplementary material for: The contribution of stigma to the transmission and treatment of tuberculosis in a hyperendemic indigenous population in Brazil
Source: PLoS One. 2020 Dec 16;15(12):e0243988. doi: 10.1371/journal.pone.0243988 (PMC7743939; doi:10.1371/journal.pone.0243988)
Supplement: S3 Appendix — (DOCX) [file pone.0243988.s003.docx]

**S3 Appendix. Thematic framework for analysis.**

| **Categories** | **Themes** | **Sub-themes** | **Codes** |
| --- | --- | --- | --- |
| Perceptions of TB transmission | How do you get TB? | Hunger | Malnutrition |
|  |  |  | Unclean food |
|  |  | Alcohol/drug abuse | Drugs |
|  |  |  | Alcohol |
|  |  |  | Going hungry because of abuse |
|  |  | Exposure to the elements | Wind |
|  |  |  | Cold |
|  |  |  | Insufficient protection (no shoes/clothes) |
|  |  | Uncleanliness/contamination | Unclean food |
|  |  |  | Dirty house |
|  |  |  | Dirty utensils |
|  |  |  | Dust |
|  |  |  | Ashes |
|  |  |  | Insects |
|  |  | Broken body | Violence |
|  |  |  | Accidents |
|  |  | Spells/witchcraft | Spells in the air |
|  |  |  | Fearing the disease |
|  |  |  | Disconnect between body and soul |
|  |  |  | Weak faith |
|  | How is TB transmitted from one person to another? | Weakening of the body | ‘Jumps into you’ |
| Stigma | Community/public stigma | Alcohol/drug abuse | Not caring about your appearance |
|  |  |  | Not cleaning |
|  |  |  | No eating |
|  |  |  | Leaving children hungry |
|  |  |  | Violence |
|  |  | Poverty | No shoes |
|  |  |  | No food on the table |
|  |  | Inability to take care of oneself | Alcoholism |
|  |  |  | Drug abuse |
|  |  |  | Unemployment |
|  |  |  | Passivity |
|  |  |  | Irresponsibility |
|  |  | Responsibility for transmission | Not seeking treatment/treatment delay |
|  |  |  | Disorganised |
|  |  |  | Reckless behavior |
|  |  |  | Stigma |
|  |  |  | LTBI stigma/family |
|  | Anticipated stigma | Employment | Fixed term contracts |
|  |  |  | Lack of employment |
|  |  |  | Mechanization of sugarcane sector |
|  |  |  | Need to stay in team to get the next contract |
|  |  |  | Treatment delay |
|  |  | Perceptions of TB at work | Not strong enough to handle the work after TB |
|  |  |  | Still infectious after treatment |
|  |  |  | Sharing barracks with team members |
|  |  |  | Sharing meals with team members |
|  |  |  | Treatment delay |
|  |  | Exclusion from community | Gossip |
|  |  |  | Avoidance |
|  |  |  | Rejection by extended family |
|  |  |  | Rejection at place of worship |
|  |  |  | Not invited to drink *tererê* |
|  |  | LTBI treatment of contacts | Family to go through stigmatization |
|  |  |  | Treatment delay |
|  | Experienced/enacted stigma | Experiences of stigma | Household |
|  |  |  | Extended family |
|  |  |  | Workplace |
|  |  |  | Separate utensils |
|  |  |  | Sleeping separately |
|  |  |  | Avoidance |
|  |  |  | Rejection |
|  |  |  | Lifting of isolation |
|  |  |  | Healthcare workers |
|  |  |  | LTBI treatment |
|  |  |  | DOTS treatment |
|  |  |  | Religious community |
|  |  | Enacted stigma | Separate meals for patient |
|  |  |  | Separate housing for patient |
|  |  |  | Masks |
|  |  |  | Separate utensils |
|  |  |  | Washing things separately |
|  |  |  | Will not meet patient |
|  |  |  | Will not meet relatives |
|  |  |  | Will not let patient into home/near children |
|  | Internalized/self-stigma | Self-worth | Unable to work |
|  |  |  | Less strong |
|  |  |  | Feeling strange |
|  |  | Suicidal thoughts | Rejection from family |
|  |  | Shame | Not wearing mask in public |
|  | Secondary stigma | LTBI treatment | Perceived as infectious |
|  |  |  | DOTS |
|  |  | Religious community | Exclusion |
|  |  |  | Inclusion |
|  |  | Family outside of household | Not being invited |
|  |  |  | Avoidance |
|  |  | Gossip | Being infectious |
